# Clinical presentation and management of nephrotic syndrome in the first year of life: A report from the Pediatric Nephrology Research Consortium

**DOI:** 10.3389/fped.2022.988945

**Published:** 2022-09-14

**Authors:** Alexandru R. Constantinescu, Tej K. Mattoo, William E. Smoyer, Larry A. Greenbaum, Jianli Niu, Noel Howard, Melissa Muff-Luett, Elizabeth B. Benoit, Avram Traum, Ali A. Annaim, Scott E. Wenderfer, Emilee Plautz, Michelle N. Rheault, Robert L. Myette, Katherine E. Twombley, Yu Kamigaki, Belkis Wandique-Rapalo, Mohammad Kallash, Tetyana L. Vasylyeva

**Affiliations:** ^1^Integrated Medical Sciences, Charles E Schmidt College of Medicine at Florida Atlantic University, Boca Raton, FL, United States; ^2^Pediatric Nephrology, Joe DiMaggio Children's Hospital, Hollywood, FL, United States; ^3^Pediatrics and Urology, Wayne State University School of Medicine, Detroit, MI, United States; ^4^Center for Clinical and Translational Research, The Ohio State University, Columbus, OH, United States; ^5^Pediatric Nephrology, Emory University, Atlanta, GA, United States; ^6^Department of Research and Scholarly Activity at Memorial Healthcare System, Hollywood, FL, United States; ^7^Health Sciences Center School of Medicine, Texas Tech University, Amarillo, TX, United States; ^8^Pediatric Nephrology, Children's Hospital and Medical Center, Omaha, NE, United States; ^9^Boston Children's Hospital, Boston, MA, United States; ^10^Pediatric Nephrology, Boston Children's Hospital, Boston, MA, United States; ^11^Pediatric Nephrology, Children's Hospital of Atlanta, Atlanta, GA, United States; ^12^Pediatric Nephrology, Texas Children's Hospital, Houston, TX, United States; ^13^University of Minnesota, Minneapolis, MN, United States; ^14^Pediatric Nephrology, University of Minnesota Masonic Children's Hospital, Minneapolis, MN, United States; ^15^Pediatric Nephrology, Children's Hospital of Eastern Ontario and Ottawa Hospital Research Institute, Ottawa, ON, Canada; ^16^Pediatric Nephrology, Medical University of South Carolina College of Medicine, Charleston, SC, United States; ^17^Joe DiMaggio Children's Hospital, Hollywood, FL, United States; ^18^Pediatric Nephrology, Nationwide Children's Hospital, Columbus, OH, United States; ^19^Pediatric Nephrology, Health Sciences Center School of Medicine, Texas Tech University, Amarillo, TX, United States

**Keywords:** nephrectomy, renal replacement therapy, infantile, congenital, thromboprophylaxis, secondary immune deficiency, nephrotic syndrome (NS)

## Abstract

**Background and objectives:**

Nephrotic syndrome (NS) in the first year of life is called congenital (CNS) if diagnosed between 0–3 months, or infantile (INS) if diagnosed between 3–12 months of age. The aim of this study was to determine if there were clinically meaningful differences between CNS and INS patients, regarding clinical presentation, management and outcomes.

**Design, setting, participants, and measurements:**

Eleven Pediatric Nephrology Research Consortium sites participated in the study, using IRB-approved retrospective chart reviews of CNS and INS patients born between 1998 and 2019. Data were collected on patient characteristics, pertinent laboratory tests, provided therapy, timing of unilateral/bilateral nephrectomy and initiation of renal replacement therapy (RRT).

**Results:**

The study included 69 patients, 49 with CNS and 20 with INS, with a median age at diagnosis of 1 and 6 months, respectively. Management for the two groups was similar regarding nutrition, thyroxin supplementation, immunoglobulin administration, and thrombosis prophylaxis. Within the first 2 months after diagnosis, daily albumin infusions were used more often in CNS *vs*. INS patients (79 *vs*. 30%; *p* = 0.006), while weekly infusions were more common in INS patients (INS *vs*. CNS: 50 *vs*. 3%; *p* = 0.001). During the 6 months preceding RRT, albumin infusions were more frequently prescribed in CNS *vs*. INS (51 *vs*. 15%; *p* = 0.007). Nephrectomy was performed more often in CNS (78%) than in INS (50%; *p* = 0.02). End-stage kidney disease tended to be more common in children with CNS (80%) *vs*. INS (60%; *p* = 0.09).

**Conclusion:**

Compared to INS, patients with CNS had a more severe disease course, requiring more frequent albumin infusions, and earlier nephrectomy and RRT. Despite center-specific variations in patient care, 20–40% of these patients did not require nephrectomy or RRT.

## Introduction

Nephrotic syndrome (NS) is the most common glomerular disease in children, but its occurrence in the first year of life is rare. It is defined as congenital nephrotic syndrome (CNS) if the disease onset is in the first 3 months of life, or infantile nephrotic syndrome (INS) if it occurs between 3 and 12 months of life. CNS, if left untreated, is a fatal disease (1, 2) but over the past four decades novel treatment approaches have greatly enhanced the survival of these patients (among them: nutritional support, attempts to prevent infections and thromboses, use of anti-proteinuric agents, refined methods of renal replacement therapy and renal transplantation at a younger age) (3–5). Management is challenging and influenced by many factors, including the degree of edema and the associated complications caused by massive proteinuria and hypoalbuminemia.

Treatment may include the use of anti-proteinuric agents such as renin-angiotensin-aldosterone system (RAAS) inhibitors (6) and nonsteroidal anti-inflammatory drugs (NSAIDs - e.g., indomethacin) (7), intravenous albumin infusions with or without administration of diuretics, and aggressive nutritional supplementation (130 kcal/kg/day).

Due to the consequences of urinary losses of specific proteins, many patients receive specific interventions, including L-thyroxine supplementation (8, 9), medications to decrease the risk of thrombosis (10, 11), and intravenous or subcutaneous immunoglobulin (IVIG/SCIG). Potentially life-threatening infections due to impaired immunity and central lines for albumin infusions, must be treated promptly.

Many patients eventually require bilateral nephrectomy followed by dialysis until transplantation (2, 12) in order to decrease the risk of infections and blood clots (1). Unilateral nephrectomy is an alternative to bilateral nephrectomy, while decreasing the amount of protein losses, obviating the need for immediate dialysis (6, 8, 13). Anecdotal reports about the management of these children without nephrectomy reflect a wide spectrum of clinical presentations and treatment strategies (5).

The aims of our study were: 1) to describe and compare the clinical presentation of children with CNS and INS, and 2) to investigate practice patterns for their management across North America.

## Methods

### Patients

Eleven sites, members of the Pediatric Nephrology Research Consortium (PNRC), participated in a retrospective chart review of patients born between 1998 and 2019. IRB approval was obtained at each participating site. Study inclusion criteria were a diagnosis of CNS or INS. Infectious etiologies (RPR positive, Hepatitis B/C, etc.) or immune-mediated nephritides, with or without hypocomplementemia, were excluded.

### Data collection

A secure, 21.CFR compliant tool was created in Qualtrics for data entry. The data collected included age at diagnosis; serum creatinine at various time points, as eGFR is not standardized for children in the first year of life (14); supportive measures; the type and timing of nephrectomy; the timing of renal replacement therapy (RRT) initiation; as well as the approach to RRT with respect to dialysis or pre-emptive kidney transplantation.

### Statistical analysis

Patient demographics and clinical characteristics were reported as counts and proportions for categorical variables and medians with interquartile ranges (IQRs) or means with standard deviations for continuous variables, as appropriate. Differences between patients' characteristics and management with CNS and INS were determined by the Mann-Whitney *U* test or Welch's *t-*test for continuous variables and by the chi-square or Fisher exact test for categorical variables, as appropriate. Counts and frequencies of missing values of the two groups were displayed. No imputation was made for missing data. Statistical analysis was performed with Prism version 9.0 statistical software. A 2-sided *p* < 0.05 indicates statistical significance. Kaplan-Meier estimates were generated, and a log-rank test was performed to calculate the probability of requiring nephrectomy (Nx) or RRT during the 2 years following diagnosis of CNS or INS.

## Results

The study included data from 69 children, 49 with CNS (59% females) and 20 with INS (55% females).

### Baseline characteristics

The median age at diagnosis was 1 month for CNS (IQR 1–1) and 6 months for INS (IQR 5–7.8). Genetic testing was performed in 50 (72%) patients, 39 with CNS, and 11 with INS, *p* = 0.04. Negative results were noted in two patients, and an additional one result was inconclusive (94% positivity rate). Two-thirds of the patients had documented edema, half were noted as having anasarca. Patients with INS had a higher serum albumin concentration at the time of diagnosis compared to CNS, with no difference in serum creatinine (Table 1).

**Table 1:** Baseline characteristics in children with CNS and INS.

**Characteristics**	**Total (*n* = 69)**	**CNS (*n* = 49)**	**INS (*n* = 20)**	***P* Value**
Age at Dx, months				
Median (IQR)	1 (1-4)	1 (1-1)	6 (5-7.8)	<0.001
Gender				
Female	40 (58)	29 (59)	11 (55)	0.75
Genetic testing (positive 94%)	50 (72)	39 (80)	11 (55)	0.04
Edema	44 (64)	30 (61)	14 (70)	0.49
Anasarca	24 (55)^a^	15 (50)^a^	9 (64)^a^	0.25
Serum albumin at Dx (g/dL)				
Median (IQR)	1.4 (1.0–1.6)	1.3 (1.0–1.6)	1.6 (1.3–1.9)	0.05
Missing values	11 (16)	11 (22)	0 (0)	0.03
Serum creatinine at Dx (g/dL)				
Median (IQR)	0.30 (0.20–0.40)	0.30 (0.20–0.50)	0.28 (0.20–0.33)	0.20
Missing values	9 (13)	9 (18)	0 (0)	0.05

CNS, Congenital nephrotic syndrome; INS, Infantile nephrotic syndrome; Dx, diagnosis; IQR, Interquartile range.

a , The denominator is the number of patients with edema.

### Treatments and complications

About a third of all patients received high energy (130 kcal/kg/day) and high protein (3–4 g/kg/day) diets. This intervention was more common in children with CNS (Table 2).

**Table 2:** Interventions in children with CNS and INS.

**Intervention**	**Total** ** (*n* = 69)**	**CNS** ** (*n* = 49)**	**INS** ** (*n* = 20)**	***p* Value**
Nutrition				
High energy diet (130 kcal/kg/day)	21 (30)	18 (37)	3 (15)	0.09
Missing values	1	1	0	
High protein diet (3–4 g/kg/day)	24 (35)	19 (39)	5 (25)	0.27
Albumin infusion				
Albumin infusions during 6 m after Dx	44 (64)	34 (69)	10 (50)	0.13
Frequency of infusions during first 2 m after Dx	44 (100)	34 (100)	10 (100)	0.13
Twice a day	1 (2)	1 (3)	0 (0)	0.99
Daily	30 (68)	27 (79)	3 (30)	0.006
Every other day	2 (5)	1 (3)	1 (10)	0.41
Three times a week	5 (11)	4 (12)	1 (10)	0.99
Weekly	6 (14)	1 (3)	5 (50)	0.001
Albumin during 6 m prior to RRT	28 (41)	25 (51)	3 (15)	0.007
Anti-proteinuric therapy				
RAAS inhibition	54 (78)	38 (78)	16 (80)	0.99
NSAID^a^ use	19 (28)	16 (33)	3 (15)	0.23
RAAS inhibitor + NSAID	18 (26)	15 (31)	3 (15)	0.24
Nephrectomy	48 (70)	38 (78)	10 (50)	0.02
Unilateral, preceding RRT	7 (15)	5 (13)	2 (20)	0.99
IVIG/SCIG treatment	16 (23)	14 (29)	2 (10)	0.12
L-thyroxine	48 (70)	36 (73)	12 (60)	0.27

Data was presented as *n* (%) of total or subset. NB: 2 patients (5 m and 48 m) with CNS - unilateral nephrectomy, RRT not initiated prior to reporting. NSAID^a^: the sole NSAID reported to be used as indomethacin. RRT, renal replacement therapy; m, month(s); Dx, diagnosis; RAAS, renin-angiotensin-aldosterone system; NSAID, nonsteroidal anti-inflammatory drug; IVIG, intravenous immunoglobulin; SCIG, subcutaneous immunoglobulin.

Albumin infusions were prescribed during the first 6 months following diagnosis in 64% of all patients, 34 with CNS and 10 with INS. Of note, in the first 2 months after establishing the diagnosis, albumin infusions were prescribed daily in 27 patients with CNS and only in 3 patients with INS (*p* = 0.006), whereas weekly infusions were reported in only one infant with CNS *vs*. 5 with INS (*p* = 0.001). The significant difference in albumin infusion requirements was maintained over the course of the disease, being higher in those with CNS than in those with INS in the 6 months preceding RRT, 25 with CNS *vs*. 3 with INS (*p* = 0.007).

RAAS inhibition was prescribed in 54 patients (78%), 38 with CNS, and 16 with INS. Of those with CNS, 22 received albumin infusions during the 6 months preceding RRT, compared to 3 with INS (χ^2^ = 4.3022; *p* = 0.038).

Indomethacin, the sole NSAID used, was prescribed in 19 patients, 16 with CNS, of which 15 also received RAAS inhibitors, and 3 with INS, who also received RAAS inhibition. Indomethacin was discontinued in 6 patients (32%) due to adverse effects: AKI (*n* = 5) and epistaxis (*n* = 1).

IVIG or SCIG was prescribed in 16 patients (23%), including 14 with CNS and 2 with INS (Supplementary Figure 3A, *p* = 0.124). Four patients with CNS (29%) receiving IVIG/SCIG experienced sepsis, compared to none in the INS group (Supplementary Figure 3B, *p* = 0.125; also Supplementary Table 4). The timing of sepsis in relationship to immunoglobulin administration could not be determined from the data available.

L-thyroxin supplementation was reported in 70% of patients, all due to abnormal thyroid function tests, without differences between CNS and INS (Table 2).

There were 12 thrombotic events reported in 7 patients with CNS, and 7 events in 3 patients with INS. Locations included venous and arterial vessels and the right atrium (Table 3). Among the children with CNS, 21 (43%) were reported to have received thrombosis prophylaxis from diagnosis, with single or multiple agents, and 7 patients experienced thrombotic events. In infants with INS, 8 (40%) received prophylaxis from the moment diagnosis was made, and 3 patients experienced thrombotic events. Thrombotic events were comparable in patients with CNS who received thrombosis prophylaxis *vs*. those who did not (4.8 *vs*. 21.4%; *p* = 0.2145). Similar finding was observed in patients with INS who received thrombosis prophylaxis *vs*. those who did not (0 *vs*. 21.4%; *p* = 0.5211). Also, for patients who received thrombosis prophylaxis, the thrombotic events were comparable in patients with CNS *vs*. those with INS (4.8 *vs*. 0%; *p* > 0.99).

**Table 3:** Thrombosis prophylaxis and incidence in CNS/INS patients.

	**CNS (*n =* 49)**	**INS (*n =* 20)**	***p* Value**
Thrombosis prophylaxis started at Dx	21 (43)	8 (40)	0.25
LMWH	16 (76)^a^	4 (50)^a^	0.21
Aspirin	3 (14)^a^	2 (25)^a^	0.59
Warfarin	1 (5)^a^	0 (0)^a^	0.99
LMWH + Aspirin	0 (0)^a^	2 (25)^a^	0.07
Warfarin + Aspirin	1 (5)^a^	0 (0)^a^	0.99
Total thrombotic events	12	7	
Initial (patients *vs*. “n” patients)	7 (14)	3 (15)	0.99
No prophylaxis	6 (86)	3 (100)	0.99
On prophylaxis	1 (14)	0 (0)	0.99
Recurrence (events)	5	4	
Treatment and prophylaxis after initial thrombosis			
LMWH	4 (57)^b^	1 (33)^b^	0.99
LMWH + Aspirin	0 (0)^b^	1 (33)^b^	0.30
LMWH + Aspirin + Warfarin	1 (14)^b^	0 (0)^b^	0.99
Thrombectomy + LMWH	1 (14)^b^	0 (0)^b^	0.99
Unknown	1 (14)^b^	0 (0)^b^	0.99
Thrombus location			
Venous	9 (75)^c^	7 (100)^c^	0.26
Arterial	2 (16)^c^	0 (0)^c^	0.51
Right atrium	1 (9)^c^	0 (0)^c^	0.99

Data presented as number (percentage) of patients or thrombotic events [*n* (%)]. LMWH, low molecular weight heparin; Dx, diagnosis; CNS, congenital nephrotic syndrome; INS, infantile nephrotic syndrome; Percentages calculated using

a : the total number of CNS or INS patients reported on thrombosis prophylaxis from Dx as the denominator;

b : the total number of CNS or INS patients with thrombosis as the denominator;

c : the total number of the thrombotic events in each group as the denominator.

**Figure 1:**
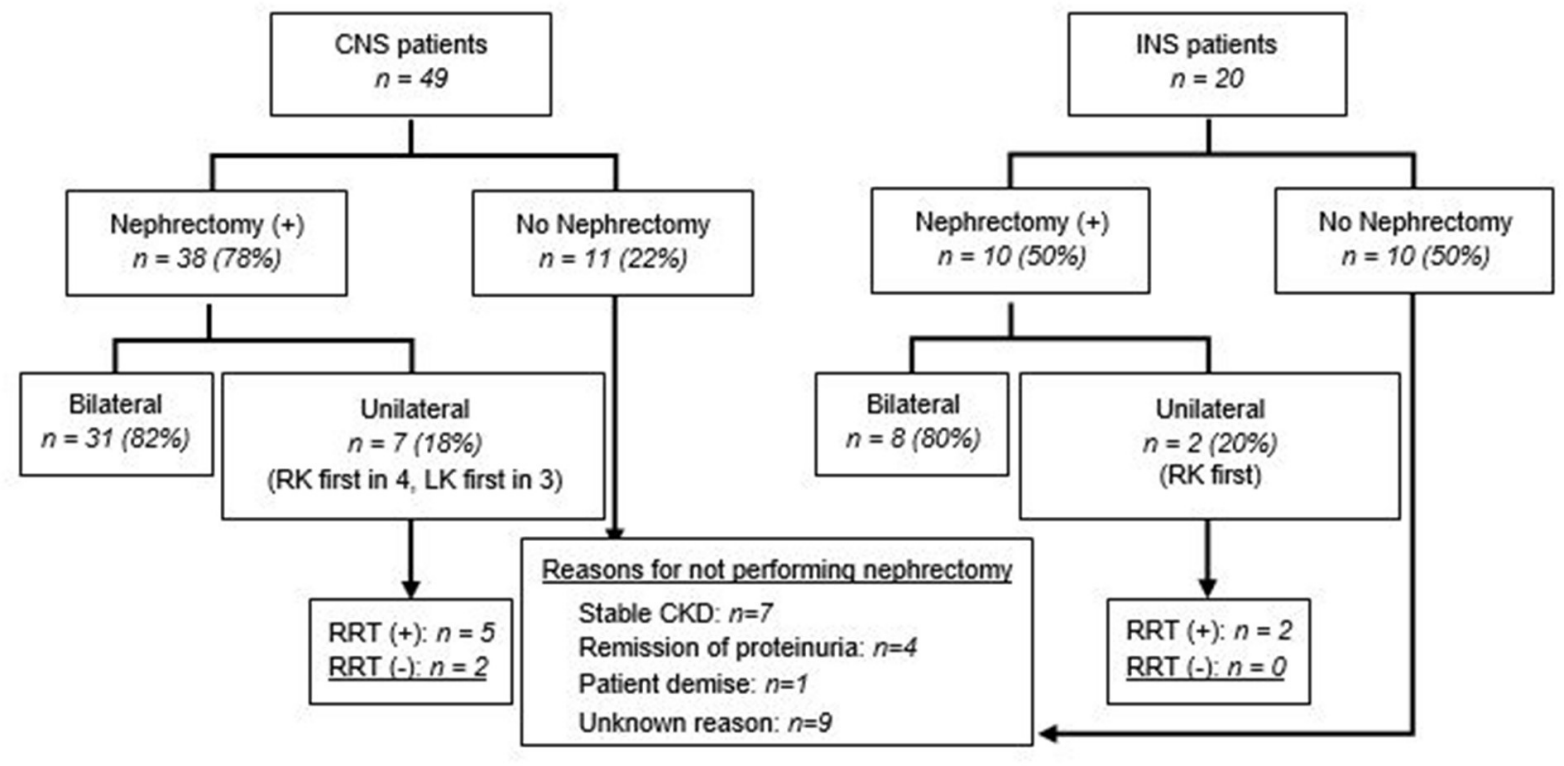
Nephrectomy in patients with CNS and INS. Unilateral nephrectomy includes sequential nephrectomy. RK, right kidney; LK, left kidney.

**Figure 2:**
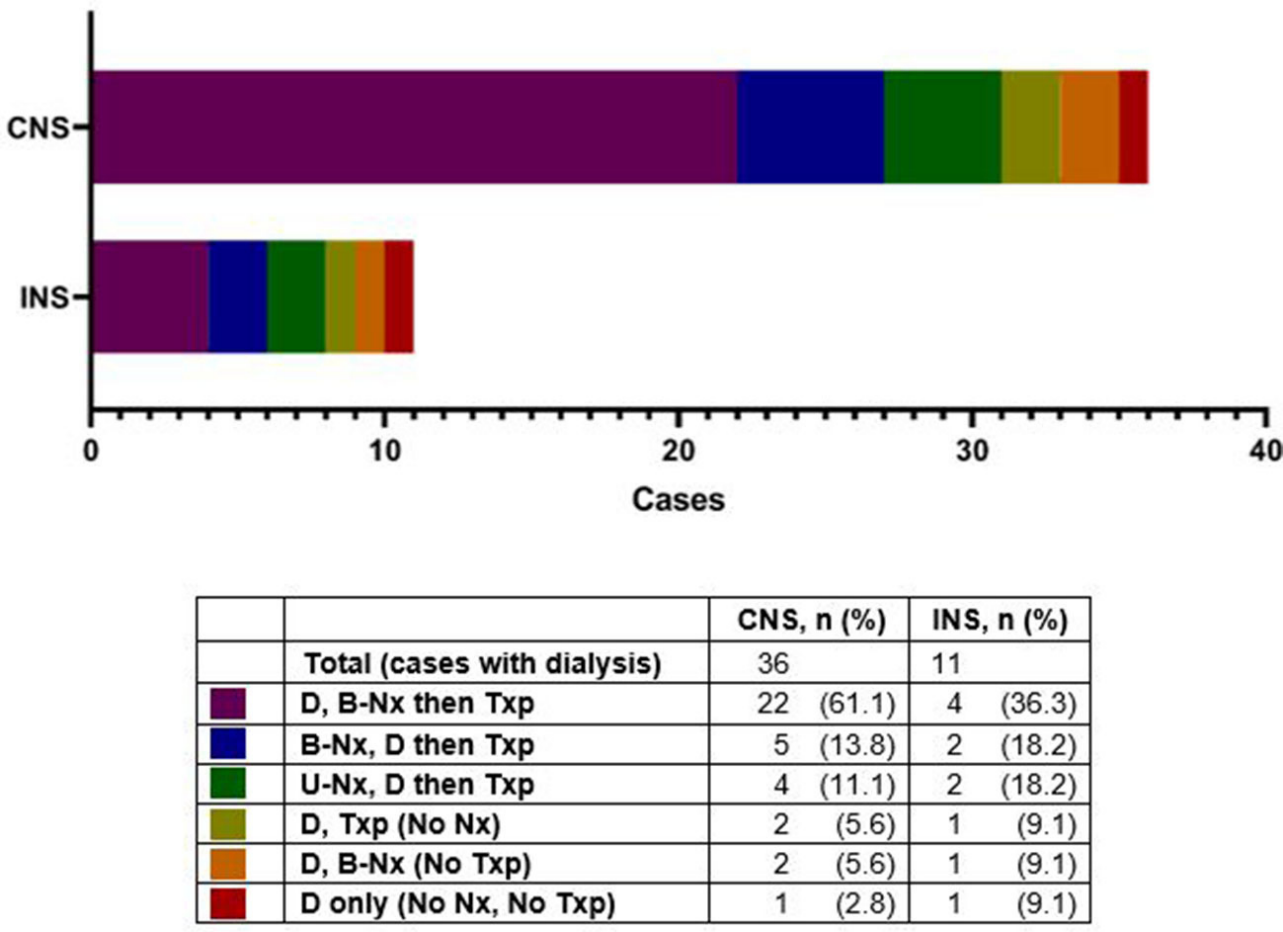
Dialysis in patients with congenital nephrotic syndrome (CNS) and infantile nephrotic syndrome (INS), with various intervention sequences. D, dialysis; B-Nx, bilateral nephrectomy; UNx, unilateral nephrectomy; Txp, kidney transplant.

Nephrectomy was performed in 48 (70%) patients, with a higher rate in infants with CNS compared to those with INS (38 *vs*. 10; *p* = 0.02). Nephrectomy was unilateral, with no RRT at the time of reporting in 2 (4%), unilateral/sequential, preceding RRT, in 7 (15%), and bilateral in 39 (81%), most often after dialysis, or at the initiation of dialysis (Table 2 and Figures 1, 2). The median age for unilateral and bilateral nephrectomy was 4 months (range 2–11 months) and 16 months (range 3–109 months), respectively.

**Figure 3:**
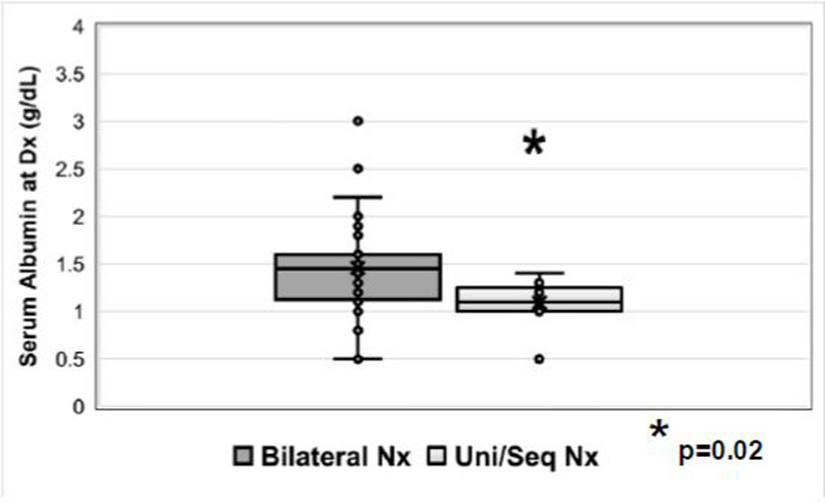
Serum albumin (g/dL) at the time of diagnosis in patients who underwent bilateral nephrectomy or unilateral/sequential nephrectomy. Nx=nephrectomy.

Of patients with unilateral or unilateral/sequential nephrectomy, eight (8/9; 89%) received RAAS inhibitors and one (1/9; 11%) received indomethacin for anti-proteinuric effect. The median serum albumin in those with unilateral/sequential nephrectomy was 1.1 g/dL (range 0.5–1.4 g/dL) *vs*. 1.5 g/dL (range 0.5–3.0 g/dL) in those with bilateral nephrectomy (*p* = 0.02) (Figure 3, Supplementary Figure 1). Histopathology results were not requested as part of this study.

Twenty-one patients (30%) did not undergo nephrectomy due to remission of proteinuria (2 spontaneous; 2 after steroid therapy, both with INS), patient demise (*n* = 1), stable CKD (*n* = 7), or unknown reasons (*n* = 9) (Figure 1). The retrospective nature of the study did not allow us to retrieve details on the steroid/other immunosuppressant response beyond those entered by the centers' research teams. With respect to the patients who achieved remission after steroid therapy, one patient was a 6 month-old who achieved remission after steroid therapy, had a kidney biopsy revealing diffuse mesangial proliferation, without sclerosis, and was being managed as steroid-sensitive idiopathic NS, without nephrectomy or RRT. The other patient was a 7 month-old, who on the kidney biopsy had diffuse mesangial proliferation with podocyte fusion and rare segmental sclerosis, and after 3 years of follow-up remains in remission, without nephrectomy or RRT. None of them underwent genetic testing. Details about patients who underwent unilateral/sequential nephrectomy, or no interventions, are presented in Supplementary Tables 1–3. As with the timing for nephrectomy, center-to-center variability was also noted regarding dialysis initiation (Figure 2 and Supplementary Table 3). Most patients with CNS (61%) had dialysis followed by bilateral nephrectomy and renal transplantation.

**Figure 4:**
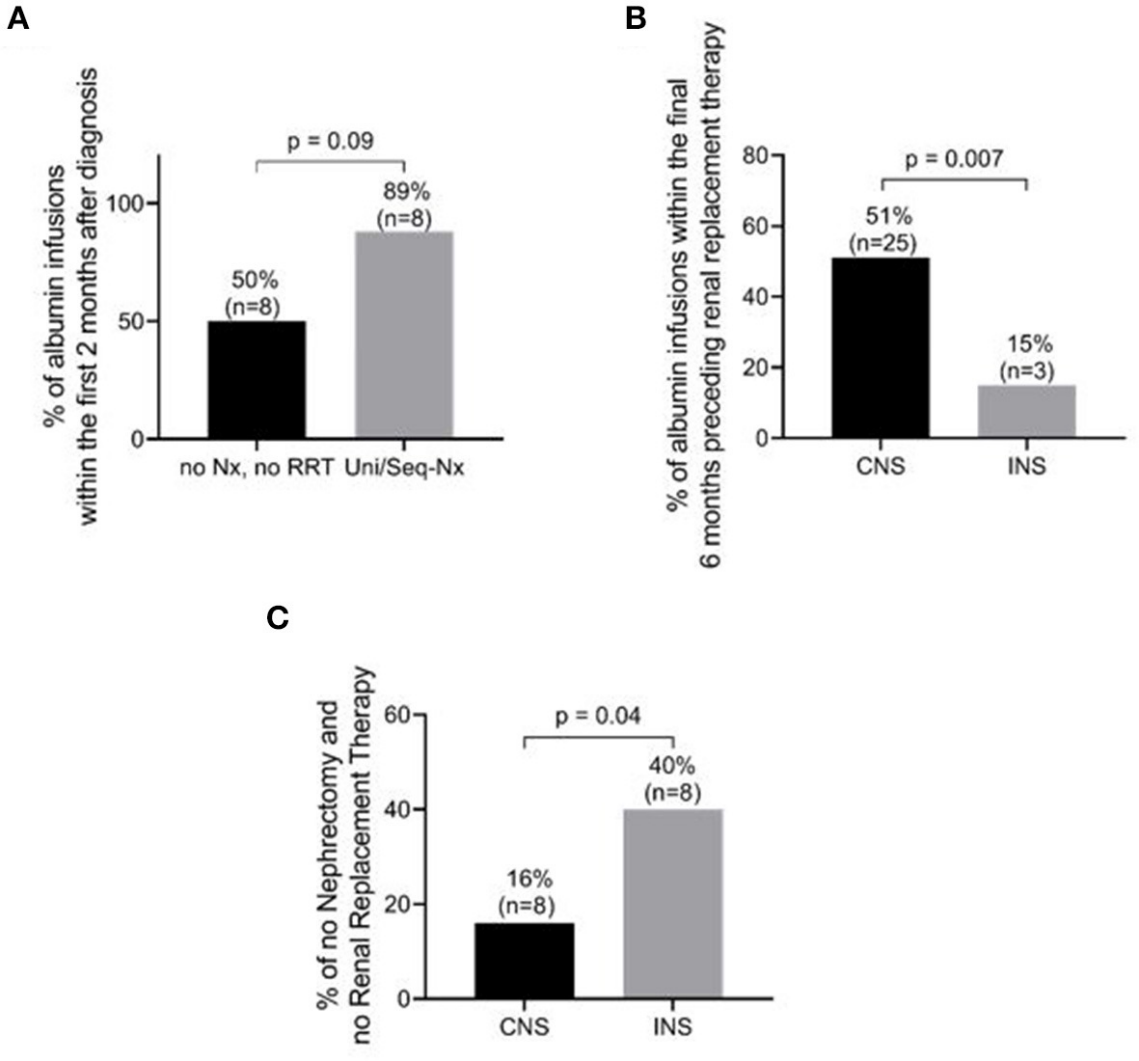
Management and outcome differences between patients with congenital nephrotic syndrome (CNS) and those with infantile nephrotic syndrome (INS). **(A)** Percentage of patients requiring albumin infusions within the first 2 months after diagnosis in CNS+INS patients. Patients with unilateral/sequential nephrectomy tended to receive more frequent albumin infusions than patients without nephrectomy and RRT (*P* = 0.09) **(B)** Percentage of patients requiring albumin infusion within the 6 months preceding RRT. CNS patients were more likely to require albumin infusions (*p* = 0.007). **(C)** Percentage of patients without nephrectomy and without renal replacement therapy (RRT). INS patients required fewer interventions than CNS patients (*p* = 0.04). See details in text and Table 2 regarding frequency of infusions. Nx, nephrectomy; Uni/Seq-Nx, unilateral/sequential nephrectomy.

RRT consisted of dialysis in 47 patients (68%), 36 with CNS and 11 with INS (73 *vs*. 55%; *p* = 0.09), and pre-emptive kidney transplantation in 4 children (3 with CNS and 1 with INS) (Table 4). Eight of the 49 children with CNS (16%) did not undergo either nephrectomy or RRT, compared to 8 of 20 (40%) with INS (χ^2^ = 4.47; *p* = 0.04) (Figure 4C).

**Table 4:** Outcomes in children with CNS and INS.

**Outcomes**	**Total (*n* = 69)**	**CNS (*n* = 49)**	**INS (*n* = 20)**	***p* Value**
RRT	51 (74)	39 (80)	12 (60)	0.09
Breakdown of RRT				
Dialysis	47 (68)	36 (73)	11 (55)	0.14
Age, months	16 (1–109)^†^	16 (1–109)^†^	18 (3–59)^†^	0.60
Subsequent transplantation	42 (89)^a^	33 (92)^a^	9 (82)^a^	0.08
No transplantation yet	5 (11)^a^	3 (8)^a^	2 (18)^a^	0.64
Pre-emptive transplantation	4 (6)	3 (6)	1 (5)	0.99
Age, months	70.5 (51–85)^‡^	77.0 (44.5–93)^‡^	64	
Time to RRT, months	17.0 (7–39.5)^‡^	16.0 (6–37.5)^‡^	18.5 (10.8–43.8)^‡^	0.58
RRT at 12 months post Dx	21 (30)	15 (31)	6 (30)	0.96
Dialysis	20 (95)^b^	14 (93)^b^	6 (100)^b^	0.91
Pre-emptive transplant	1 (5)^b^	1 (7)^b^	0 (0)^b^	0.99
Serum Albumin (g/dL)				
At 12 months post Dx	2.6 ± 1.2	2.6 ± 1.3	2.7 ± 1.0	0.48
Missing values	16 (23)	12 (24)	4 (20)	0.76
At RRT	2.1 ± 0.7	2.1 ± 0.7	2.1 ± 0.7	0.96
Missing values	30 (43)	19 (39)	11 (55)	0.22

Data available was presented as n (%) of total or of specific therapy, median (

† : range,

‡ : Interquartile range) or mean ± SD. RRT, renal replacement therapy; Dx, diagnosis; N/A, data not available. Percentages were calculated using

a : the number of patients with dialysis as the denominator, and

b : the number of patients with RRT at 12 months post Dx as the denominator.

Dialysis was initiated at a median age of 16 months (range 1–109) for the CNS group and 18 months for the INS group (range 3–59) followed by transplantation in 42 patients (89% of those requiring dialysis, at the time of reporting) (Table 4). The time to RRT was 17 months (IQR 7–39.5), with no significant difference between CNS and INS, 16 (IQR 6–37.5) and 18.5 (IQR 10.8–43.8), respectively (*p* = 0.58).

Pre-emptive kidney transplantation was performed in 3 patients with CNS *vs*. 1 with INS, at a median age of 70.5 months (IQR 51–85).

### Outcomes

In patients with unilateral/sequential nephrectomy, 89% (8/9) required albumin infusions during at least the first 2 months following diagnosis, compared to only 50% (8/16) of those patients who did not undergo nephrectomy or RRT (*p* = 0.09) (Figure 4A and Table 5A). Of the 39 patients with CNS who underwent RRT (36 dialysis and 3 pre-emptive kidney transplant), 25 (64%) received albumin infusions during the 6 months preceding RRT, 3 (14%) of which did not undergo nephrectomy (Figure 4B and Table 5B). In comparison, of the 12 patients with INS who underwent RRT (11 dialysis and one pre-emptive kidney transplant), only 3 (25%) received albumin during 6 months prior to RRT (*p* = 0.007, Table 5B). Eight of 49 children with CNS did not require nephrectomy or RRT, compared to 8 of 20 (40%) with INS (χ^2^ = 4.47; *p* = 0.04). RAAS inhibition was recorded in 8 of the 10 patients (80%) with CNS *vs*. 6 of the 8 patients (75%) with INS.

**Table 5A:** Albumin infusions in children with CNS and INS who did not require interventions (Nx or RRT) *vs*. those who underwent Unilateral/Sequential nephrectomy.

	**Total**	**No Nx, No RRT (*n =* 16)**	**Uni/Seq Nx (*n =* 9)**	***p* Value**
Albumin infusions during first 2 months after Dx	25	8 (50)	8 (89)	0.09

Data available was presented as n (%) of total. CNS, congenital nephrotic syndrome; INS, infantile nephrotic syndrome; Nx, nephrectomy; RRT, renal replacement therapy; Uni/Seq Nx, unilateral/sequential nephrectomy.

**Table 5B:** Albumin infusions in children with CNS and INS.

**Outcomes**	**Total (*n* = 69)**	**CNS (*n* = 49)**	**INS (*n* = 20)**	***p* Value**
No Nx, No RRT	16 (23)	8 (16)	8 (40)	0.035
Albumin infusions during 6 months preceding RRT	28 (41)	25 (51)	3 (15)	0.007

Data available was presented as n (%) of total. CNS, congenital nephrotic syndrome; INS, infantile nephrotic syndrome; Nx, nephrectomy; RRT, renal replacement therapy; Dx, diagnosis.

The outcome at 12 months (Table 4) was not different between the two groups with respect to serum albumin concentration, or RRT requirement.

## Discussion

The age-based definitions of CNS and INS have been used for decades to help guide the diagnosis, management and predict clinical outcomes in patients with NS in the first year of life. More recently, however, this rationale is being questioned because of increasing knowledge about the genetic basis of early-onset NS including the fact that a particular gene defect can present as CNS or INS (15). Although genetic testing is now widely available and could significantly predict disease severity and prognosis, only 55% of children with INS in our study were tested over the years for specific mutations. A somewhat higher percentage of infants with CNS (80%) were genetically tested. This low percentage of genetic testing probably reflects the high costs and difficulties of obtaining genetic testing in North America, especially during the earlier years of this cohort. In the future, we would expect genetic testing to become a diagnostic standard for CNS and INS, as genetic *vs*. non-genetic etiologies of NS in the first year of life require a more precise diagnosis.

Several management approaches have been recommended. These include albumin infusions, NSAIDs and/or RAAS inhibitors, nephrectomy, and RRT (6, 12). In addition, some patients exhibit secondary hypothyroidism and immune deficiency, which may be treated with L-thyroxin and immunoglobulin infusions, respectively. Last, but not least, thrombosis prophylaxis needs to be considered.

Our study revealed a considerable variation in the management of CNS and INS among North American centers, and even among patients at the same center. The rarity of the disease does not help consolidate single-center clinical experience or study the management outcomes, difficult tasks for all rare disorders, requiring personalized approach to care. Along with the variations in clinical presentation, physicians' personal beliefs and preferences play a role in management, in the absence of any evidence-based guidelines. Facing the same challenges, the European Reference Network for Kidney Diseases (ERKNet) and the European Society for Pediatric Nephrology (ESPN) Working Group recently published their expert opinion-based consensus guidelines for the management of CNS (16).

Our study, which is the largest such study to date in North America, revealed that at the time of diagnosis, edema was present in two-thirds of patients, at similar rates in CNS and INS. The question of why the remainder of the patients with congenital nephrotic syndrome did not have clinically evident edema in the setting of low serum albumin, is not completely understood, although interstitial fibrosis with tubular atrophy along with low eGFR could have been responsible for the lack of edema in some patients (3, 7). Our data provide evidence-based support to the recommendation that albumin infusions should be prescribed based on clinical indicators (i.e., anasarca, ascites, sepsis, thrombotic events), not solely the serum albumin concentrations.

Since the only available data are the number of patients who received RAAS inhibitors and NSAIDS, known as anti-proteinuric agents, and the protein/creatinine ratio is less reliable at this age because of low urine creatinine, we could not evaluate the impact of these medications on proteinuria in this special population.

We also found that 50% of those with CNS required albumin infusions during the 6 months preceding RRT, compared to 15% of patients with INS, although there was no significant difference in the use of NSAIDs and RAAS inhibitors, or unilateral nephrectomy, between the groups. Although it is unclear if this approach postponed nephrectomy or renal replacement therapy, or led to a complication-free course, patients with INS who received RAAS inhibitors were less likely to receive albumin infusions during the 6 months prior to RRT when compared to those with CNS.

Historically, the lack of minimal response to steroid therapy in this patient population has shifted the treatment paradigm toward symptomatic relief with emphasis on optimal nutrition and growth, until the moment RRT is needed. In our study, we have identified only 2 children with INS who were prescribed steroid therapy, because genetic testing was not available at that time. The kidney biopsy, performed as indicated by the lack of response after the conventional timeframe, identified minimal change disease in one and mesangial hypercellularity with sclerosis in the other, and was reassuring that both achieved remission. As the availability of genetic testing is increasing, steroid therapy or other immunosuppressive drugs will likely be prescribed in those cases with no identified gene mutations, who on biopsy will be found to have minimal change or focal segmental glomerulosclerosis.

Although well justified, supplementation with high caloric diet was received by only a third of the patients in our study. We believe that a dietitian should be part of a complex management team for these patients, and more attention should be paid to CNS and INS infants' nutrition to improve their growth.

With the goal to diminish proteinuria, NSAIDs and RAAS inhibitors were widely used by many centers. In our study, RAAS inhibitors were prescribed alone or in addition to indomethacin to prevent inadequate growth and development from the massive urine protein losses. Indomethacin had to be stopped in a third of those receiving it, due to side effects. The addition of anti-proteinuric agents has helped a significant number of these children to require less often albumin infusions in the 6 months preceding RRT, more so in INS than in CNS. However, due to the various lengths of follow-up and timing to RRT, we cannot determine the exact impact of these agents on the renal survival in this cohort. Nonetheless, based on our data, we support the initiation of therapy with low-dose anti-proteinuric agents, titrating to effect, monitoring for side effects, and not exceeding the maximal dose.

Prevention of infection is an important part of the treatment approach for CNS and INS patients. The urinary losses of immunoglobulin and complement factors, along with the use of indwelling catheters, increase the risk for bacterial infections in these infants. There is a paucity of data on the effectiveness of IVIG/SCIG for preventing infections in these patients. In 21 infants with CNS due to pathogenic variants in *NPHS1* (frequently called “Finnish type NS”), with a median follow-up of 1.1 years, one study reported 63 verified, and 62 suspected, episodes of sepsis (17). In a French cohort (18), the use of central venous lines was associated with an increase in rates of staphylococcal bacteremia. Neither the use of prophylactic antibiotics nor prophylactic immunoglobulin infusions were associated with reduced frequency of infections (5, 12, 17, 18). In a multicenter European cohort of CNS patients with *NPHS1* mutations there was also no significant difference in the rates of peritonitis (32 and 13%), central line infections (48 and 47%), or sepsis (54 and 53%) between 17 patients managed conservatively and 25 patients with bilateral nephrectomy, 2.8 years after diagnosis (19, 20). Infection rates were also similar before and after initiation of dialysis. Mortality from infectious causes has been reported in 5–10% of patients (17, 18). The consensus recommendations of the ERKNet-ESPN Working Group did not include recommendations on prophylactic antibiotics or IV immunoglobulin (16). Even though we did not collect data on serum IgG concentration, or response to immunizations, infants with nephrotic syndrome are considered immune-deficient, and prescription of IVIG/SCIG was at the discretion of the treating team. In our North American cohort, IVIG or SCIG appeared to be protective, though decision-making should be individualized until more data become available.

Thromboembolic events are a known complication in NS, occurring more often in adults than in children with NS from various etiologies, as the result of an imbalance of anti-thrombotic and pro-thrombotic factors, favoring thrombosis (11). Our study found that those patients on routine prophylaxis, using a variety of agents, had a reduced rate of thrombosis (Table 3). There have been data showing that the degree of hypercoagulability is proportional to the degree of disease activity in patients with biopsy proven NS (21, 22). The ERKNet-ESPN Working Group has recommended consideration of routine anticoagulant use in children with CNS and/or prior thrombosis (16). Some patients with thrombosis experienced recurrences despite initiation of prophylaxis. Thus, high-risk patients may need combination prophylaxis therapy very early on in the course of the disease. Future studies to elucidate the most appropriate use of thrombosis prophylaxis are clearly needed.

Infants with NS often have low serum thyroxine (T4) level, attributed mostly to the urinary loss of thyroid-binding globulin and elevated thyroid-stimulating hormone (TSH), with improving levels following thyroxine replacement (8, 23). Routine thyroid screening and early replacement therapy has become the standard of care in such patients, knowing that TSH normalizes later than T4 (16, 24). Our data confirmed the need for thyroid-replacement in the majority of patients with CNS and INS. It is interesting to note that hypothyroidism may persist after bilateral nephrectomy, even after kidney transplant, raising the possibility of an intrinsic thyroid disorder in some patients (9). In those cases, pediatric endocrinology input is necessary.

In 2019, the ESPN Dialysis Working Group published their findings on 80 children with CNS (19), 55% of whom required dialysis before age 2, 93% being placed on peritoneal dialysis. Some patients, particularly those with CNS, underwent bilateral nephrectomy for intractable proteinuria non-responsive to conservative management (4, 25). The sole objective was to stop proteinuria and alleviate complications associated with severe hypoproteinemia, including growth failure. Bilateral nephrectomy may also be done in preparation for a renal transplant and in patients with Denys-Drash syndrome because of a high risk for Wilms' tumor. Unilateral nephrectomy (6, 13, 26) along with anti-proteinuric agents is a viable alternative to bilateral nephrectomy (27–29), the major advantage being that it may decrease or stop the need for albumin infusions and facilitate outpatient management without an immediate need for RRT. However, in some patients, unilateral nephrectomy may not be enough, and second nephrectomy followed by dialysis may be needed (Supplementary Table 3). As of now, there are no known markers to help differentiate between the patients who may do better with unilateral as compared to the bilateral nephrectomy. In the present study, the lower median serum albumin concentration might explain the need for unilateral nephrectomy at an earlier age as compared to the bilateral nephrectomy, especially in those with CNS. Dufek et al. (20) have described a step-wise approach, as an alternative to early nephrectomy and renal replacement therapy. More detailed studies about growth, blood pressure and nutritional status, among other parameters, will be needed to determine the benefits of unilateral *vs*. bilateral nephrectomy *vs*. early renal replacement therapy in these rare genetic disorders.

In patients with *NPHS1* mutations (18), the survival and complication rates (peritonitis, central line infections, septic episodes, thrombotic events, height SDS) were not different in patients who were treated with bilateral nephrectomy and RRT, compared to patients who underwent conservative treatment. Timing of RRT appears not to influence the survival and growth in congenital NS caused by mutations in *NPHS1*, although pre-emptive renal transplantation was not reported in CNS of Finnish type (30). Our data also showed that a large number of patients were managed successfully without the need for RRT. Genetic testing will be pivotal in assessing the outcome related to various pathogenic mutations, not only those of *NPHS1*.

The unilateral or sequential nephrectomy was justified and completed in those patients who during the first 2 months after diagnosis required frequent albumin infusions. Therefore, the need for daily albumin infusions, and treatment with RAAS inhibitors and/or NSAIDs that is ineffective or leads to complications, is a reasonable indication for unilateral nephrectomy. On a case-by-case basis, pre-emptive renal transplantation should be discussed with the patient's family.

In summary, our data confirmed that patients with CNS have an earlier presentation with lower serum albumin concentrations compared to INS and a more severe disease course, requiring earlier nephrectomy and/or RRT. However, many infants with CNS and INS have been able to be successfully managed for a prolonged time without RRT.

Our study has some limitations: a) the voluntary participation does not allow us to draw the conclusion that the cohort is representative of nephrotic syndrome in the first year of life in the North American population, as there are centers caring for patients such as these, not members of PNRC, and member-centers of PRNC who did not participate in this study; b) its retrospective design does not allow us to determine outcomes related to certain therapies; c) the few missing data prevented us from reaching firm conclusions in some areas. At the same time, our study has some strengths: a) to date, it is the largest cohort of nephrotic syndrome in the first year of life in North America; b) provides data supporting the majority of the prior expert recommendations.

Based on the data described, we are proposing the following diagnostic and management guidelines:

Genetic testing should become a diagnostic standard for suspected CNS and INS.

A high-energy diet (130 kcal/kg/day) is an essential component of the management of CNS and INS, and a dietitian should be part of the treatment team.

Albumin infusions should be based on clinical indicators, not serum albumin concentration.

Therapy with RAAS inhibitors and NSAIDs should be initiated at a low dose and titrate to effect with close monitoring of side effects, not exceeding the maximal dose.

The need for prophylaxis of thromboembolic events should be made on a case-by-case basis, mostly after thrombotic events or other thrombophilia indications.

Thyroid replacement therapy is justifiable in the complex management of CNS and INS patients.

Prevention of infection is an important part of the treatment approach for CNS and INS patients. There is a lack of strong data supporting or denying IV or SC immunoglobulin infusions, and this therapy should be decided individually.

Patients with *WT1* mutation are appropriate candidates for early bilateral nephrectomy.

Future studies should include the creation of a prospective worldwide registry to include the diagnosis, management, complications, and long-term outcomes of infants with CNS and INS to better enable pediatric nephrologists to develop robust evidence-based approaches to improve the care of these critically ill infants.

## Data availability statement

The original contributions presented in the study are included in the article/Supplementary material, further inquiries can be directed to the corresponding author.

## Ethics statement

The studies involving human participants were reviewed and approved by institution-specific IRBs. Written informed consent from the participants' legal guardian/next of kin was not required to participate in this study in accordance with the national legislation and the institutional requirements.

## Author contributions

AC, TV, TM, LG, and WS: conceptualization. AC, NH, MM-L, EB, EP, MR, BW-R, and MK: data curation. AC: formal analysis and writing-original draft. TV, AC, and JN: methodology. WS and TV: supervision. TM, WS, JN, YK, and TV: validation. YK: visualization. SW, AA, AT, MR, RM, LG, KT, TV, TM, and WS: writing-review and editing. All authors contributed to the article and approved the submitted version.

## Funding

Texas Tech University Health Sciences Center Collaborative Seed Grant provided support for TV.

## Conflict of interest

The authors declare that the research was conducted in the absence of any commercial or financial relationships that could be construed as a potential conflict of interest.

## Publisher's note

All claims expressed in this article are solely those of the authors and do not necessarily represent those of their affiliated organizations, or those of the publisher, the editors and the reviewers. Any product that may be evaluated in this article, or claim that may be made by its manufacturer, is not guaranteed or endorsed by the publisher.
