# Supplementary material for: Clinical presentation and management of nephrotic syndrome in the first year of life: A report from the Pediatric Nephrology Research Consortium
Source: Front Pediatr. 2022 Sep 14;10:988945. doi: 10.3389/fped.2022.988945 (PMC9534228; doi:10.3389/fped.2022.988945)

<u>Supplemental material - Table of Content:</u>

Supplemental Table 1. Age at diagnosis of congenital nephrotic syndrome (CNS) and infantile nephrotic syndrome (INS), with frequency of albumin infusions during the first 2 months after diagnosis (Dx) in those without intervention (nephrectomy or renal replacement therapy).

Supplemental Table 2. Timing of nephrectomy (Nx) and frequency of albumin infusions in patients with congenital nephrotic syndrome (CNS) and infantile nephrotic syndrome (INS) who underwent unilateral/sequential nephrectomy.

Details about unilateral/sequential nephrectomy

Supplemental Table 3. Serum albumin and timing of 2nd nephrectomy, type of and age at renal replacement therapy (RRT) in patients with congenital nephrotic syndrome (CNS) and infantile nephrotic syndrome (INS) who underwent unilateral/sequential nephrectomy.

Supplemental Table 4. Use of intravenous/subcutaneous immunoglobulin (IVIG/SCIG) in patients with congenital nephrotic syndrome (CNS) and infantile nephrotic syndrome (INS), as well as number of patients with sepsis while on IVIG/SCIG.

Supplemental Figure 1. Serum albumin (g/dL) at diagnosis and 12 months later in patients with congenital nephrotic syndrome (CNS) and infantile nephrotic syndrome (INS) who underwent bilateral nephrectomy (Bi-Nx) *vs*. unilateral/sequential nephrectomy (Uni/Seq-Nx).

Supplemental Figure 2. Probability of requiring nephrectomy (Nx) or renal replacement therapy (RRT) during the 2 years following diagnosis of congenital nephrotic syndrome (CNS) and infantile nephrotic syndrome (INS)

Supplemental Figure 3. Use of IVIG/SCIG for infection prophylaxis. (A) IVIG/SCIG CNS *vs*. INS patients. (B) Sepsis in CNS *vs*. INS patients. IVIG, intravenous immunoglobulin; SCIG, subcutaneous immunoglobulin; CNS, congenital nephrotic syndrome; INS, infantile nephrotic syndrome

Supplemental Table 1. Age at diagnosis of CNS and INS, with frequency of albumin infusions (number of infusions per month; #/m) during the first 2 months after Dx in those without intervention (nephrectomy or renal transplant therapy).

Patient ID	Age at Dx (m)	Serum Albumin at Dx (g/dL)	Edema at Dx	Frequency of Albumin infusions 2 months after Dx (#/m)	Observation Period (months)[a]
CNS – 1	1	2.3	None	60	21
CNS – 2	3	N/A	Anasarca	30	35
CNS – 3	1	N/A	N/A	30	104
CNS – 4	1	0.9	Edema	30	12
CNS – 5	1	0.2	N/A	30	12
CNS – 6	1	2.3	None	0	78
CNS – 7	1	0.6	Anasarca	0	60
CNS – 8	1	1.6	Edema	0	1
INS – 1	4	3.6	None	0	3
INS – 2	4	3.2	None	0	68
INS – 3	6	3.5	None	0	15
INS – 4	6	1.8	Anasarca	4	57
INS – 5	6	2.3	None	0	56
INS – 6	6	1.6	Anasarca	0	N/A
INS – 7	7	1.5	Anasarca	0	11
INS – 8	10	1.7	Anasarca	4	36

CNS, congenital nephrotic syndrome; INS, infantile nephrotic syndrome; m, month; Dx, diagnosis; N/A, data not available. a: This was calculated using the dates of presentation and last visit.

Supplemental Table 2. Timing of nephrectomy and frequency of albumin infusions in patients with CNS and INS who underwent unilateral/sequential nephrectomy. Age at RRT as reference

Patient ID	Age at Dx (m)	Age at 1st Nx (m)	Age at 2nd Nx (m)	Age at RRT (m)	Frequency of Albumin Infusions 2 m after Dx (#/m)	Albumin Infusions 6m prior to RRT	Observation Period[a] (m)
CNS – 9	1	1	109	109	30	No	
CNS – 10	1	2	-	-	30	No (No RRT)	5
CNS – 11	2	3	5	3	30	Yes	
CNS – 12	1	4	6	6	30	Yes	
CNS – 13	1	4	6	6	30	Yes	
CNS – 14	2	4	-	27	30	No	
CNS – 15	1	8	-	-	12	No (No RRT)	48
INS – 9	9	11	18	18	4	No	
INS – 10	5	5	-	19	0	No	

CNS, congenital nephrotic syndrome; INS, infantile nephrotic syndrome; Dx, diagnosis; m, month(s); RRT, renal replacement therapy; 1st Nx, first nephrectomy; 2nd Nx, second nephrectomy; [a]: Observation period of patients without RRT.

Details about unilateral/sequential nephrectomy in patients with CNS and INS

Of patients with unilateral or unilateral/sequential nephrectomy, eight (8/9; 89%) received RAAS inhibitors and one (1/9; 11%) received indomethacin for anti-proteinuric effect.

Two patients with CNS underwent unilateral nephrectomy without dialysis or transplantation.

Four patients with CNS and one with INS underwent unilateral nephrectomy followed by removal of the second kidney at the time of dialysis, with subsequent kidney transplantation.

One patient with CNS underwent unilateral nephrectomy with the second nephrectomy at the time of pre-emptive transplant, and did not receive dialysis.

Three out of these seven patients with CNS needed sequential nephrectomy, while no patient with INS needed a sequential nephrectomy.

One patient with INS had a unilateral nephrectomy, then dialysis followed by a transplant.

Three patients, two with CNS and one with INS underwent nephrectomy at the time of pre-emptive transplantation.

Unilateral nephrectomy was not helpful in 3 (33%) of 9 patients, and they needed second nephrectomy and RRT soon thereafter. Six patients (67%) did not have the second nephrectomy or RRT at the time of reporting, after a median follow-up period of 18.5 months (range 3-105).

Supplemental Table 3. Serum albumin and timing of 2nd nephrectomy, type of and age at RRT in patients with CNS and INS who underwent unilateral/sequential nephrectomy.

Patient ID	Age (m), Alb at Dx (g/dL)	Edema at Dx	Age (m), Alb at 12 m (g/dL)	Alb at 2nd Nx (g/dL), Type of RRT, Age (m) at RRT
CNS-9	1, 1.2	Anasarca	13, 3.6	3.3, at pre-emptive txp, 109 m
CNS-10	1, 0.5	Edema	13, N/A	N/A (no 2nd Nx), no RRT at time of report
CNS-11	1, 1.1	Edema	13, 3.4	N/A, 2nd Nx was 2 m later from 1st Nx and RRT, 3 m
CNS-12	1, 1.0	Anasarca	13, N/A	N/A, 2nd Nx and Dialysis, 6 m
CNS-13	1, 1.0	Anasarca	13, N/A	N/A, 2nd Nx and Dialysis, 6 m
CNS-14	2, 1.2	Anasarca	14, 1.4	N/A (no 2nd Nx), Dialysis, 27 m
CNS-15	1, 1.3	Edema	13, 1.6	N/A (no 2nd Nx), no RRT at time of report
INS-9	9. 1.4	Anasarca	21, 4.2	1.7, 2nd Nx and Dialysis, 18 m
INS-10	5, N/A	Anasarca	17, 2.0	N/A (2nd Nx), Dialysis, 19 m

CNS, congenital nephrotic syndrome; INS, infantile nephrotic syndrome; Alb, serum albumin; Dx, diagnosis; m, month(s); RRT, renal replacement therapy, either dialysis or pre-emptive kidney transplantation; Nx, nephrectomy; N/A, data not available; Txp, kidney transplant.

Supplemental Table 4. Use of intravenous/subcutaneous immunoglobulin (IVIG/SCIG) in patients with congenital nephrotic syndrome (CNS) and infantile nephrotic syndrome (INS), as well as number of patients with sepsis while on IVIG/SCIG.

	CNS (n=49)	INS (n=20)
IVIG/SCIG	14	2
Sepsis while on IVIG/SCIG	4	0

Supplemental Figure 1. Serum albumin (g/dL) at diagnosis and 12 months later in patients with CNS and INS who underwent bilateral nephrectomy (Bi-Nx) *vs*. unilateral/sequential nephrectomy (Uni/Seq-Nx).

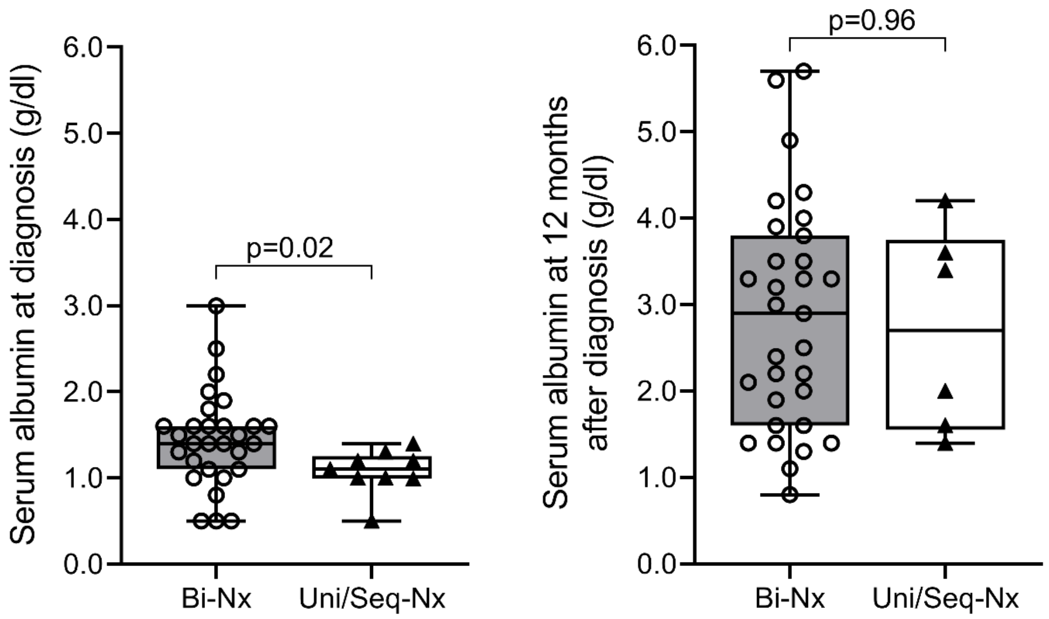

Supplemental Figure 2. Probability of requiring nephrectomy (Nx) or RRT during the 2 years following diagnosis of CNS or INS.

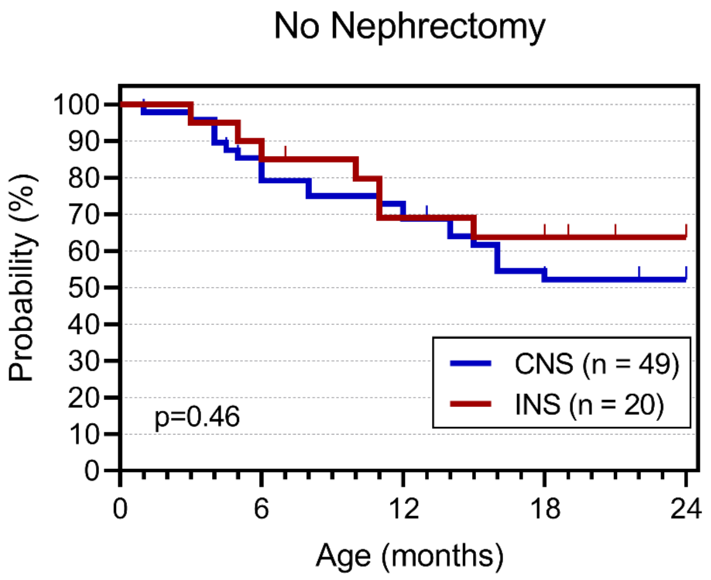

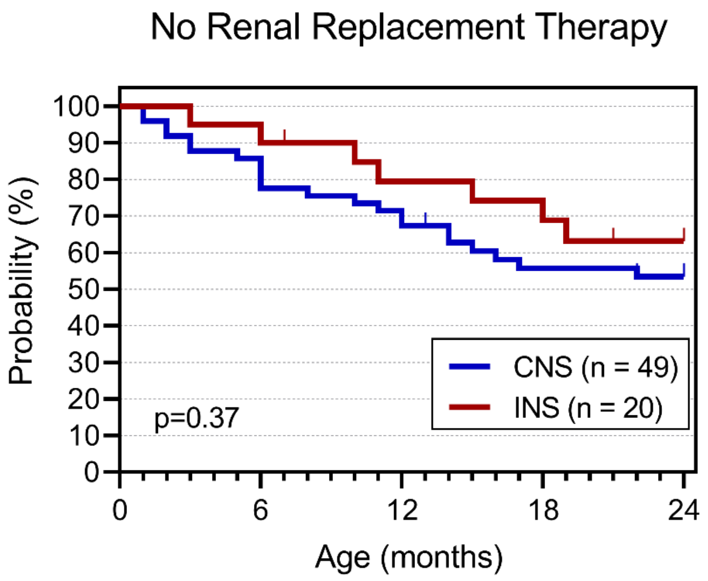

Supplemental Figure 3. Use of IVIG/SCIG for infection prophylaxis. (A) IVIG/SCIG in CNS *vs*. INS patients. (B) Sepsis in CNS *vs*. INS patients. IVIG, intravenous immunoglobulin; SCIG, subcutaneous immunoglobulin; CNS, congenital nephrotic syndrome; INS, infantile nephrotic syndrome.

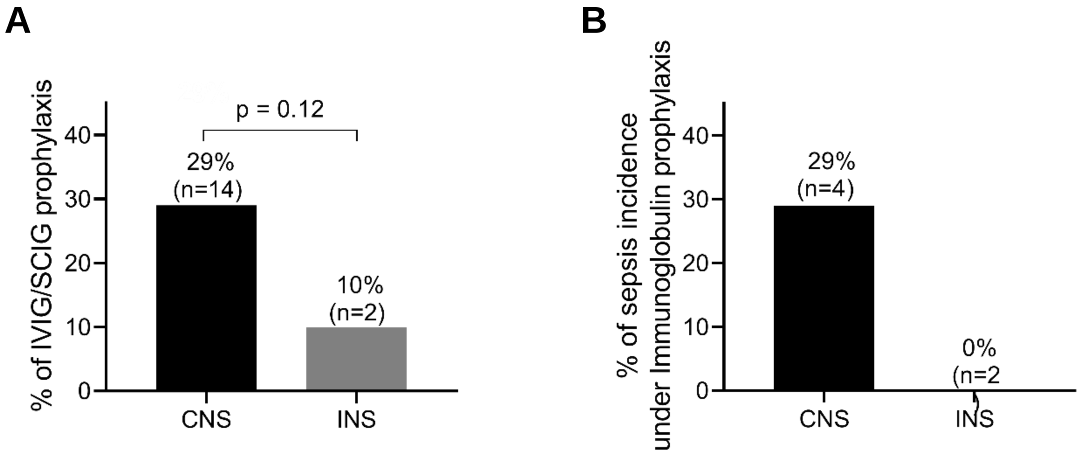

Supplement: Supplementary file 1 [file Data_Sheet_1.PDF]
